# Inclusion of Wheat Dried Distillers’ Grains with Solubles from Bioethanol Plants in Diets for Dairy Cows

**DOI:** 10.3390/ani11010070

**Published:** 2021-01-02

**Authors:** Philip C. Garnsworthy, Michael Marsden, Jennifer R. Goodman, Neil Saunders

**Affiliations:** 1 School of Biosciences, Sutton Bonington Campus, University of Nottingham, Loughborough LE12 5RD, UK; Jennifer.Hankin@nottingham.ac.uk (J.R.G.); neil.saunders@nottingham.ac.uk (N.S.); 2 AB Agri Limited, 64 Innovation Way, Peterborough Business Park, Lynch Wood, Peterborough PE2 6FL, UK; michael.marsden@abagri.com

**Keywords:** dairy cow, milk production, protein feeds, wheat-based dried distillers grains with solubles

## Abstract

**Simple Summary:**

There are environmental concerns about feeding imported soya bean meal to dairy cows in Europe. An alternative protein source is dried distillers’ grains with solubles (DDGS), a co-product of bioethanol manufacture. Corn is the main source of bioethanol and DDGS in the USA, and corn DDGS is widely researched. Wheat is used for bioethanol and DDGS manufacture in Canada and Europe, but most studies of wheat DDGS in dairy diets have used one dietary inclusion level. Responses of dairy cows to inclusion level of wheat DDGS made in Europe are unknown. In this study, we tested two batches of wheat DDGS from UK bioethanol plants, which replaced soya and rapeseed meal in diets for high-yielding dairy cows. One batch of wheat DDGS had a low proportion of solubles, which decreased its metabolisable energy content and limited inclusion level to below 20% of diet dry matter before dry matter intake and milk yield were depressed. The other batch of wheat DDGS had a typical proportion of solubles, resulting in higher metabolisable energy content, and could be included to at least 22.5% of diet dry matter without affecting dry matter intake and milk yield. Results of this study give confidence that wheat DDGS produced in Europe can be used at high inclusion levels in diets for high-yielding dairy cows.

**Abstract:**

Dried distillers’ grains with solubles (DDGS) from bioethanol production can replace soya in diets for dairy cows, but the optimum inclusion level of European wheat DDGS (wDDGS) is unknown. Two batches of wDDGS from different UK bioethanol plants were fed to 44 (Experiment 1) and 40 (Experiment 2) cows in a Latin square design. Each wDDGS replaced soya and rapeseed at four inclusion levels (g/kg of diet dry matter (DM): 0, 80, 160 and 240—Experiment 1; 0, 75, 150 and 225—Experiment 2). Diets were balanced for metabolisable energy (ME) and protein (MP), and for minimum starch and saturated fat in Experiment 2. In Experiment 1, DM intake (29 kg/day) and milk yield (42.3 kg/day) were unaffected by wDDGS inclusion up to 160 g/kg but were lower than control with 240 g/kg inclusion, which was attributed to the low proportion of solubles in this wDDGS batch. In Experiment 2, DM intake (22.4 kg/day) and milk yield (32.1 kg/day) were unaffected by wDDGS inclusion up to 225 g/kg. ME content of wDDGS, determined in vivo (MJ/kg DM) was 12.1 (Experiment 1) and 13.4 (Experiment 2). It is concluded that the optimum inclusion level of wDDGS is at least 225 g/kg DM in diets balanced for minimum starch and saturated fat as well as ME and MP supplies.

## 1. Introduction

Distillers’ grains are a co-product of fermenting and distilling cereal grains to produce alcohol, and are widely fed to animals, especially ruminants. During manufacture, starch is converted to alcohol, so the co-product has a low concentration of fermentable carbohydrates, but higher concentrations of protein, oil, fibre and ash than the original grains. Greater use of co-products in diets for dairy cows, particularly when replacing imported soya bean meal and cereals, will lower the carbon footprint of dairy products, and improve food security [1]. Historically, the main source of distillers’ grains was distilleries producing whisky and other potable spirits. Typically, distillers’ grains are mixed with liquid residues from the first distillation step (pot ale syrup), and dried to make dried distillers’ grains with solubles (DDGS). In the USA, proportions of solid and liquid components are controlled by legislation, but such restrictions do not apply in Europe [1]. Consequently, DDGS from whisky distilleries can be of variable composition, both between distilleries and between batches within a distillery. Variable composition limits inclusion levels of DDGS from whisky distilleries in animal diets [1]. 

In recent decades, DDGS have become available as co-products from distilleries producing bioethanol as a renewable fuel. The USA is the largest exporter of DDGS, exporting 8 to 12 million tonnes annually to over 50 countries between 2015 and 2019, including 0.5 to 1.0 million tonnes to the European Union [2]. The USA produces bioethanol and DDGS almost exclusively from corn (maize), and some countries (e.g., Canada and Hungary) use significant proportions of corn, whereas other countries utilise other grains, such as wheat in Canada [3], Germany [4] and the United Kingdom (current study).

Maize DDGS (mDDGS) has been studied extensively in dairy cow diets, and inclusion levels of up to 300 g/kg of diet dry matter (DM) have been reported without affecting milk yield (see review: [5]). There have been few studies of inclusion level for wheat DDGS. In Canada, when canola was replaced by mDDGS at 200 g/kg of diet DM, a 50:50 mixture of mDDGS and wheat DDGS (wDDGS), or wDDGS at 150 g/kg of DM, all types of DDGS increased milk yield compared with canola, but there was no difference between sources of DDGS, and no effect of wDDGS inclusion level [6]. Also in Canada, wDDGS replaced canola and soya bean meal at wDDGS inclusion levels up to 200 g/kg of diet DM, and increased DM intake (DMI) and milk yield compared to control, but there was no effect of wDDGS inclusion level [7]. In Germany, wDDGS (inclusion 170 g/kg DM) replaced rapeseed meal (inclusion 150 g/kg DM) without affecting milk yield [4]. In Denmark, DDGS originating from 80% wheat and 20% triticale (inclusion 140 g/kg DM) replaced a soya bean and rapeseed meal mixture without affecting milk yield [8]. The optimum inclusion level of wDDGS produced in Europe has not been reported before the current study.

Two new bioethanol plants opened in the UK in 2010 and 2013, with a combined annual capacity to produce 850,000 tonnes of wDDGS from locally grown wheat. These plants presented an opportunity to reduce reliance on imported soya bean meal and mDDGS in diets for UK dairy cows. To maximise this opportunity, knowledge of effects of wDDGS inclusion on dairy cow performance was needed. Therefore, the main objective of this study was to measure responses to wDDGS inclusion level in diets for high-yielding dairy cows. This objective was achieved using two dairy cow experiments, each testing a different batch of wDDGS, from a different bioethanol plant, at four levels of inclusion. The hypothesis was that wDDGS could replace conventional ingredients in dairy diets without affecting cow performance, provided diets were balanced for energy and nutrient supplies. Because cows in early lactation have greater nutrient demands, a secondary objective in Experiment 1 was to test the effect of wDDGS inclusion level in early and mid-lactation. To support the findings, metabolisable energy (ME) concentration was determined in vivo, and rumen protein degradability was determined in sacco, for each batch of wDDGS (Appendix A).

## 2. Materials and Methods 

All animal work was carried out under the authority of the UK Animals Scientific Procedures Act (1986), within Project Licence numbers 30/3210 and 40/2751. Approval for the work was obtained prior to commencement, from the University of Nottingham Animal Welfare and Ethical Review Body. 

Two experiments were conducted to examine responses to the inclusion level of wDDGS in diets for high-yielding dairy cows. Each experiment used a single batch of wDDGS from a different UK bioethanol plant, which was tested at four inclusion levels. These experiments were supported by a metabolism trial using sheep fed at maintenance to determine in vivo ME content, and a rumen degradability trial using dairy cows to determine degradability characteristics of the wDDGS samples used. Details of the metabolism and degradability trials are provided in Appendix A.

### 2.1. Animals, Housing and Feeding

Holstein–Friesians cows from the University of Nottingham Dairy Centre (average annual milk yield 11,400 L/cow) were used in this study. Cows were group housed in a single pen of a freestall barn and were milked in an automatic milking station (AMS; Lely A3 Astronaut; Lely UK Ltd., Cambridge, UK). The feeding system consisted of partial-mixed rations (PMR) offered ad libitum through individual electronic feed bins (RIC Feeders; Fullwood Ltd., Ellesmere, UK), and concentrates offered in the AMS during milking.

Both experiments were conducted using a Latin square design with four inclusion levels of wDDGS as treatments. Treatments were applied to squares of four cows in four treatment periods. All cows were fed on the standard farm diet for two weeks before experiments started. In Experiment 1, 44 cows (mean parity 2.5 ± 1.34) were recruited and divided into two groups according to stage of lactation (Early lactation (Group E), 69 ± 11.5 days in milk (DIM), n = 16 cows; mid-lactation (Group M), 163 ± 38.8 DIM, n = 28 cows). Within Groups E and M, cows were blocked according calving date and milk yield during the second week of the pre-experiment period and allocated to squares of four cows at random within blocks. In this design, there were four squares of cows (four cows each square) for Group E and seven squares of cows (four cows each square) for Group M. In Experiment 2, 40 cows (mean parity 2.4 ± 1.26) were recruited, but were not grouped by stage of lactation (mean 183 ± 53.8 DIM). Cows were blocked according to calving date and milk yield during the second week of the pre-experiment period and allocated to 10 squares of four cows at random within blocks.

Treatment periods lasted four weeks in Experiment 1, but were shortened to three weeks in Experiment 2, following confirmation from analysis of Experiment 1 data that this would not affect results. Each treatment period consisted of two weeks diet adaptation and one or two weeks recording. Replication was 10 or 11 cows per inclusion level per period, giving an overall replication of 40 or 44 cows per inclusion level.

Partial-mixed rations were mixed and dispensed with an automated system (Mix Feeder and Smart Feeder; Mullerup, Ullerslev, Denmark). Forage components of the PMR were mixed first, and then concentrate blends containing all non-forage components were added (see later). Each treatment PMR was mixed and dispensed into feed bins in two batches between 07:30 and 15:30 daily. Individual cows had unrestricted access to all feed bins containing their treatment PMR, and there were between 1.4 and 1.6 cows per feed bin. Quantities of PMR mixed each day were adjusted to ensure at least 10% of PMR offered was still available next day, i.e., feeding was truly ad libitum. Cows were offered concentrates during milking at 3.5 kg per cow per day, plus an additional 0.45 kg/L of milk produced above a threshold yield, up to a maximum individual concentrate allowance of 12 kg per day or 3 kg per AMS visit. Threshold milk yields in Experiment 1 were 35 L/day for Group E, and 30 L/day for Group M. Threshold milk yield in Experiment 2 was 32 L/day for all cows. No concentrate refusals were observed, and previous studies give confidence that cows consume their entire concentrate allowance in the AMS.

### 2.2. Treatments

Treatment diets were formulated to supply energy and nutrient requirements according to the Feed into Milk system [9] for maintenance plus milk yields of 35 L/day for Group E and 30 L/day for Group M in Experiment 1, and 32 L/day for all cows in Experiment 2. Formulation of diets for these milk yield thresholds included the baseline quantity of concentrates (3.5 kg/day) fed in the AMS, which was deducted from total diet formulations in order to provide PMR formulations (Table 1).

In Experiment 1, two diets were formulated as control diets; one diet was for cows in early-lactation (Group E), and one for cows in mid-lactation (Group M). Both diets were based on grass, maize and whole-crop silages (totalling approximately 0.5 of diet DM), supplemented predominantly with soya bean meal, rapeseed meal, wheat and protected fat (Table 1). Metabolisable energy and metabolisable protein supplies of these diets were then used as constraints to formulate equivalent diets where the main protein supplement was wDDGS. Soya bean meal, rapeseed meal and protected fat were excluded from these diets, and small amounts of wheat and SoyPass were included to maintain supplies of rumen microbial protein and bypass protein. The inclusion level of wDDGS in these diets was 240 kg/t of diet on a DM basis. To provide intermediate levels of wDDGS inclusion, quantities of ingredients for diets containing wDDGS at 0 and 240 kg DM/t of diet DM were used in ratios of 2:1 and 1:2 when mixing PMRs. For early (E) and mid- (M) lactation groups, therefore, treatment diets contained wDDGS at 0, 80, 160 and 240 g DM/kg of diet DM.

In Period 2 of Experiment 1, some cows exhibited signs of mild acidosis, including lowered rumination time, loose faeces, and fluctuation in daily feed intake. This was not related to treatment diet and was attributed to change in physical structure of the grass silage between Periods 1 and 2. For Periods three and four, therefore, chopped straw was added to the PMR at the rate of 0.5 kg/cow/day to aid rumination. Proportions of other forages were adjusted to maintain inclusion levels of wDDGS.

In Experiment 2, a control diet was formulated that was similar to the control diet for Group M in Experiment 1, but with molassed sugar beet pulp and chopped straw included to increase NDF concentration. An equivalent diet was formulated where the main protein supplement was wDDGS. Soya bean meal, rapeseed meal and molassed sugar beet pulp were excluded from this diet, and small amounts of wheat and SoyPass were included to maintain supplies of rumen microbial protein and bypass protein. The inclusion level of protected fat was set equal to the control diet to maintain supply of bypass fatty acids across treatments. The inclusion level of wDDGS in this diet was 225 kg/t of diet on a DM basis. The aim of formulation in Experiment 2 was not only to balance metabolisable energy and metabolisable protein supplies across treatments, as in Experiment 1, but also to reduce contrasts in starch and NDF concentrations between diets with zero and highest inclusion levels of wDDGS. All non-forage ingredients of the two formulated PMR were blended in a single batch per PMR so that ingredient proportions would not vary during the study. To provide intermediate levels of wDDGS inclusion, blends for diets containing wDDGS at 0 and 225 kg DM/t of diet DM were used in ratios of 2:1 and 1:2 when mixing PMRs. Therefore, treatment diets contained wDDGS at 0, 75, 150 and 225 g DM/kg of diet DM.

### 2.3. Sampling and Recording

Samples of each forage and concentrate blend were taken weekly and stored at −20 °C. Samples were thawed and pooled at the end of each feeding period. Pooled samples were sent for analysis in a commercial laboratory (Sciantec Analytical, Cawood, UK). Forages were analysed using near-infrared (NIR) spectroscopy and Forage Analysis Assurance Group equations to predict nutrient contents (https://www.faagroup.co.uk). Concentrate blends and AMS concentrates were analysed using wet chemistry. Neutral Detergent Fibre (NDF) in feed samples were determined according to [10] with the use of a heat-stable α-amylase (Sigma, Gillingham, UK) with the omission of sodium sulphite. Starch was determined according to Method 996.11 of [11]. Starch hydrolysis proceeded in two phases; in phase 1 starch was partially hydrolysed and totally solubilised. In Phase 2, starch dextrins were quantitatively hydrolysed to glucose by amyloglucosidase. Complete solubilisation of starch was achieved by cooking the sample in the presence of heat-stable α-amylase. Protein (Nitrogen) content was determined using the DUMAS method (990.03; [11]) using a Leco Truspec Analyser (Leco Instruments, Stockport, UK). Metabolisable energy was estimated by applying a calculation from a variety of parameters as detailed by [12]. In addition, weekly samples of each forage were used for DM determination by oven drying at 80 °C for 48 h. Composition of PMRs is shown in Table 2.

Milk yield and live weight were recorded for each cow at each milking throughout the trial. Milk samples were collected over three to five days in the last week of each feeding period, covering all milking times throughout the day and night (i.e., two morning, two afternoon and two night samples per cow). Individual milk samples were analysed for butterfat, protein, lactose, and urea using mid-infrared spectroscopy at the National Milk Laboratories, Wolverhampton, UK. Daily mean values for milk components were calculated by weighting analytical results for milk component yield at each sampled milking as a proportion of total milk yield for all sampled milkings. Energy-corrected milk yield (ECM) was calculated as ECM (kg/day) = milk yield (kg/day) × {(38.30 × fat content (g/kg) + 24.20 × protein content (g/kg) + 16.54 × lactose content (g/kg) + 20.7)/3140} [13]. Body condition score was recorded for each cow weekly using a scale of 1 to 5 [14].

Methane emissions were recorded automatically during each milking using the online monitoring system developed at the University of Nottingham [15]. This system monitors methane concentration in the AMS feed bin at one-second intervals using a non-dispersive infrared gas analyser (Guardian, Edinburgh Instruments, Edinburgh, UK). Peaks in methane concentration due to eructations by cows are used to estimate daily methane emissions with an established calibration against respiration chambers [15].

Rumination time data were recorded throughout the trial by using sensor tags on neck collars (Lely Qwes system, Lely UK Ltd., Cambridge, UK) and downloaded during each milking. Rumination was expressed as number of minutes per day spent ruminating. In Experiment 2, rumen fluid samples were collected via stomach tube (Ruminator; www.profs-products.com) between 09:00 and 12:00 on one day in the last week of each feeding period for analysis of volatile fatty acids (VFA) [16].

Blood samples were collected between 09:00 and 12:00 on one day in the last week of each feeding period via the jugular vein. Samples were centrifuged for 10 to 15 min (3500× *g* at 4 °C), and stored at −20 °C until analysed for the following metabolites on a Bayer opera autoanalyzer (Bayer UK Ltd., Newbury, UK): non-esterified fatty acids (NEFA; Wako kit NEFA-C; Alpha Laboratories Ltd,. Eastleigh, UK), β-hydroxy butyrate (BHB; Randox kit Ranbut RB 1008; Randox Laboratories Ltd, Crumlin, UK), total protein (Bayer kit T01 130102), albumin (Bayer kit T01 137702), globulin (total protein minus albumin), urea-N (Bayer kit T01 182356) and glucose (Bayer kit T01 183356).

Faecal samples were collected from each cow between 09:00 and 12:00 at the start and end of each recording week for digestibility determination. Samples were collected by grab sampling between 09:00 and 12:00 and oven dried at 80 °C until constant weight, which was reached after 3 to 5 days. Nitrogen concentration in feed and faeces was determined using a Thermo Scientific Flash 2000 elemental analyser (Fisher Scientific UK Ltd, Loughborough, UK). Dry matter digestibility was determined from the ratio of acid insoluble ash (AIA) in feed and faeces [17]. Nitrogen digestibility was determined from ratios of AIA and N in feed and faeces.

### 2.4. Statistical Analysis

All data were calculated as daily means and averaged per individual cow over weeks three and four of each period in Experiment 1, and week three of each period in Experiment 2. Each experiment was analysed separately.

Data were analysed using Genstat (19th Edition; VSN International, Hemel Hempstead, UK). The residual maximum likelihood (REML) procedure was used to fit linear mixed models. Fixed effects in the models were: lactation stage group (E or M in Experiment 1); wDDGS inclusion level (0, 80, 160, 240 in Experiment 1; 0, 75, 150, 225 in Experiment 2); and interaction between lactation stage and wDDGS inclusion level. Random effects in the model were: square (1–11 in Experiment 1, 1–10 in Experiment 2); Period (1, 2, 3, 4); and individual cow. Inclusion level was entered in the model as a factor to examine differences between treatment means, and as a variate to determine linear and quadratic responses. There was no quadratic response to wDDGS inclusion level for any measurement, so only linear effects were retained in the model. The final model was:

*y_ijk_* = *μ* + *G_r_* + *L_s_* + *GL_rs_* + *S_i_* + *P_j_* + *C_k_* + *ε_ijk_* (1)

where *y_ijk_* is the dependent variable; the fixed part of the model consists of *μ* the overall constant (grand mean), *G_r_* the main effect of Group *r* (where *r* is the stage of lactation group for unit *ijk*; Experiment 1), *L_s_* the main effect of DDGS inclusion level *s* (where *s* is the inclusion level for unit *ijk*), and *GL_rs_* the interaction between group and the inclusion level; the random model terms are: *S_i_* the effect of Square *i*, *Pj* the effect of Period *j*, *C_k_* the effect of Cow *k*, and *ε_ijk_* the random error (i.e., residual) for unit *ijk*. Data are presented as least-square means predicted by the models for main effects (lactation stage and inclusion level), and linear effects of inclusion level. Least-square means were compared using a least significant difference test. Statistical significance was declared at *p* < 0.05, and a tendency at *p* < 0.01. 

## 3. Results

### 3.1. Experiment 1

When cows were fed on the highest wDDGS inclusion level (240 g/kg), they consumed less dry matter and PMR than when fed on 0 or 80 g/kg, but intake values for 160 g/kg were not different from other levels (Table 3). Overall, there was a linear decrease in intakes of dry matter and PMR, but not concentrates fed in the AMS. Yields of milk, ECM, fat, protein and lactose decreased with increasing wDDGS inclusion level, but treatment means were only significantly different for the comparison of 0 and 240 g/kg. There was no effect of wDDGS inclusion level on milk fat, protein, or lactose concentrations, but milk urea concentration was greater for wDDGS inclusion levels of 0 and 80 g/kg compared with 160 and 240 g/kg. Overall, there were linear decreases in milk fat and urea concentrations.

Cows in early lactation had higher intakes of dry matter and concentrate, and higher yields of milk, ECM, protein, and lactose than cows in mid-lactation (Table 3). Milk fat concentration was lower for cows in early lactation than for cows in mid-lactation, so milk fat yield did not differ between lactation stages. Milk urea concentration was higher for cows in early lactation than for cows in mid-lactation.

There was no effect of wDDGS inclusion level or stage of lactation on live weight or body condition score (Table 3). Methane output (g/day) was not affected by wDDGS inclusion level or stage of lactation. Methane yield (g/kg DMI) increased linearly with increasing wDDGS inclusion level, was lower with a wDDGS inclusion level of 80 g/kg than 240 g/kg, but was not affected by stage of lactation. Rumination time was not affected by wDDGS inclusion level but tended to be longer (*p* = 0.063) for cows in early lactation than for cows in mid-lactation. Dry matter digestibility was not affected by wDDGS inclusion level. Nitrogen digestibility decreased linearly with increasing wDDGS inclusion level, and was lower with a wDDGS inclusion level of 240 g/kg than 0 and 80 g/kg.

There was no effect of wDDGS inclusion level or stage of lactation on plasma concentrations of albumin, globulin, total protein, or glucose (Table 4). Plasma urea-N decreased with increasing wDDGS inclusion level and was higher for cows in early lactation than for cows in mid-lactation. Plasma BHB increased with increasing wDDGS inclusion level and was lower for wDDGS inclusion levels 0 and 80 g/kg than for 160 and 240 g/kg but was not affected by stage of lactation. Plasma NEFA concentration was not affected by wDDGS inclusion level but was higher for cows in early lactation than for cows in mid-lactation. There was no interaction between lactation stage and wDDGS inclusion level for any measurement.

### 3.2. Experiment 2

There was no effect of wDDGS inclusion level on dry matter intake, milk yield or milk composition, except for milk urea concentration (Table 5). Overall, however, there was a linear decrease in dry matter intake with increasing wDDGS inclusion level. Milk urea concentration decreased with increasing wDDGS inclusion level, and was lower when cows were fed wDDGS inclusion levels of 150 and 225 g/kg than when they were fed inclusion levels of 0 and 75 g/kg. There was no effect of wDDGS inclusion level on live weight, body condition score, methane, rumination time or digestibility. There was no effect of wDDGS inclusion level on any plasma metabolite, although there was a linear decrease in plasma urea-N with increasing wDDGS inclusion level (Table 6). There was no effect of wDDGS inclusion level on rumen pH, total VFA or individual VFA concentrations, except for isobutyrate (Table 7). Isobutyrate concentration decreased with increasing wDDGS inclusion level, and was lower when cows were fed wDDGS inclusion level 225 g/kg than when they were fed inclusion levels of 0 and 75 g/kg. Isovalerate concentration tended to decrease with increasing wDDGS inclusion level (*p* = 0.015), but there was no difference between treatment groups.

### 3.3. Metabolisable Energy and Protein Degradability of wDDGS

Metabolisable energy content of the batch of wDDGS used in Experiment 1 (wDDGS-1) was 12.1 MJ/kg DM and ME content of the batch used in Experiment 2 (wDDGS-2) was 13.4 MJ/kg DM (Appendix A Table A2).

At a rumen outflow rate of 0.08 per hour, protein degradability was 0.455 for wDDGS-1 and 0.568 for wDDGS-2; effective rumen degradable protein (ERDP) content was 142 g/kg DM for wDDGS-1 and 205 g/kg DM for wDDGS-2; digestible undegraded protein (DUP) was 109 g/kg DM for wDDGS-1 and 96 g/kg DM for wDDGS-2 (Appendix A Table A4).

## 4. Discussion

The main objective of this study was to measure responses to wDDGS inclusion level in diets for high-yielding dairy cows. This objective was achieved by feeding cows on diets containing wDDGS at four inclusion levels in each of two experiments. Because wDDGS displaced other dietary ingredients, there were unavoidable confounding factors that might impact production responses. This study reflects diet formulation in practice and results must be interpreted as responses to entire diets and their nutrient contents. In Experiment 1, DMI, milk yield and ECM yield were lower at a wDDGS inclusion level of 240 g/kg DM than at other inclusion levels, suggesting an upper limit between 160 and 240 g/kg DM for wDDGS inclusion without affecting performance. In Experiment 2, however, DMI, milk yield and ECM yield were not affected by wDDGS inclusion level up to 225 g/kg DM. Differences between observations in the two experiments might be explained by differences between the two batches of wDDGS and degree of balancing diet formulations.

The two batches of wDDGS were manufactured in different bioethanol plants, and wDDGS-1 was manufactured before the plant was fully optimised for wDDGS production. Proportions of grains and solubles could not be measured in the finished products but discussions with the manufacturers, visual observation, and results of laboratory analysis (e.g., lower crude protein) and degradability evaluation (e.g., lower soluble nitrogen) suggest that wDDGS-1 contained a lower proportion of solubles than wDDGS-2. Rumen protein degradability was lower for wDDGS-1 than for wDDGS-2, which is consistent with a lower proportion of solubles, but could also suggest greater heat treatment (higher temperature or longer time) during the drying stage of manufacture. Diet formulation for Experiment 1 assumed a value of 13.7 MJ/kg DM for metabolisable energy (ME) content of wDDGS. This was the average in vivo ME value of wDDGS from the whisky industry in the review by [18]. The ME content determined in the current study for wDDGS-1 was 12.1 MJ/kg DM. This is similar to published values of 12.4 MJ/kg DM for wDDGS from whisky production [19], 12.6 MJ/kg DM for wDDGS from Canadian bioethanol production [20], and 12.7 MJ/kg DM for wDDGS from European bioethanol [4]. The difference between assumed and determined ME content of wDDGS-1 would reduce diet ME content by 0.4 MJ/kg DM and daily ME intake by 9 MJ/day, which would account for much of the difference in milk yield between diets with 0 and 240 g wDDGS/kg DM. The ME content determined for wDDGS-2 (13.4 MJ/kg DM) is consistent with a greater proportion of solubles and is closer to the value of [18] that was used in diet formulation. Solubles are beneficial not only for their oil and nitrogen content, but also for their content of yeast residues, which can act as a probiotic to improve rumen bacterial growth [18].

Another difference between experiments was the approach to diet formulation. In Experiment 1, diets were formulated to supply approximately equal amounts of ME and MP according to Feed into Milk [9], whereas in Experiment 2 attention was paid also to minimum starch supply and fatty acid profile. Because most of the starch is converted to ethanol during bioethanol production, wDDGS has a very low starch content. Starch content of diets decreased with increasing wDDGS inclusion level because wDDGS replaced soya bean meal, rapeseed meal and rolled wheat. Dietary starch content is important for maintaining glucose supply and insulin status of dairy cows and has implications for metabolic health and reproduction [21,22], as well as performance. In Experiment 2, therefore, rolled wheat was included in all diets to ensure a minimum starch content of 200 g/kg DM. Further, in Experiment 1, as wDDGS inclusion level increased across diets, protected fat was progressively removed from diets to maintain dietary oil content. Although the oil content of wDDGS is higher than the original wheat (e.g., 19 versus 48 g/kg DM [20]), the fatty acid profile of wDDGS is markedly different from that of protected fat. The major fatty acids in wDDGS (g/100 g fatty acids) were linoleic (57), palmitic (21) and oleic (13) acids [23], whereas the major fatty acids in the protected fat were stearic (48) and palmitic (45) acids (www.tridentfeeds.co.uk). Palmitic and stearic acids have specific roles in metabolism of dairy cows and can enhance milk yield [24], so protected fat inclusion level was set to be the same for all diets in Experiment 2.

Cows in the early lactation group of Experiment 1 had higher milk yield with a lower fat content than cows in mid-lactation, which was as expected. Higher DMI by cows in the early lactation group was due to higher intake of concentrates rather than PMR, which can be explained by their higher concentrate allowance. A key finding is the absence of interaction between stage of lactation and wDDGS inclusion level for any parameter, which means that there is no need to adjust wDDGS levels in herds that normally feed only one diet throughout lactation.

Most studies of DDGS have examined mDDGS rather than wDDGS. The inclusion level of mDDGS in diets for dairy cows was reviewed by [5], who concluded that responses in milk production have been seen up to 300 g/kg DM. Normally, however, there is no advantage in formulating diets containing mDDGS at more than 200 g/kg DM because such diets can supply excess protein and phosphorus [5]. Use of wDDGS in diets for dairy cows was reviewed by [3] who reported that inclusion levels of between 100 to 200 g/kg DM either maintained or increased milk yield relative to control diets. Responses depended on the nature of the control diet; increased milk yield was observed when wDDGS replaced barley silage or canola meal in Canada, but not when wDDGS replaced rapeseed meal in Europe [3]. Canola meal was replaced by wDDGS at 100, 150 and 200 g/kg DM in the study of [7] and there were linear increases in DMI and milk yield with increasing wDDGS inclusion level. This is somewhat contrary to results of the current study, where there was a decrease in milk yield with the highest wDDGS inclusion level in Experiment 1 and no effect on milk yield in Experiment 2. In Experiment 1, the decrease in milk yield might be attributed partly to differences in protected fat between diets, as well as supply of fermentable carbohydrates. To our knowledge, no other study has examined the inclusion level of wDDGS from bioethanol production in a single experiment.

Milk fat, protein and lactose concentrations were unaffected by wDDGS inclusion level in either experiment, which concurs with the study of [7]. Milk urea concentration, however, decreased with increasing wDDGS inclusion level in both experiments, indicating that nitrogen was being used more efficiently for synthesis of true milk protein [21]. Decreased milk urea concentration was reflected in decreased plasma urea-N in Experiment 1, but plasma urea-N was unaffected by wDDGS inclusion level in Experiment 2. Decreases in milk and plasma urea can be ascribed to decreasing protein intake coupled with the lower ERDP content of wDDGS compared to soya bean and rapeseed meals, which would have decreased excess ERDP.

Some studies have suggested that methane emissions can be reduced by DDGS. Giger-Reverdin and Sauvant [25] reviewed methane determinations performed at the Rowett Research Institute, Aberdeen, UK, and reported that DDGS had lower methane emissions than any other concentrate ingredient. In a study which replaced barley with mDDGS in beef cattle diets, McGinn [26] observed a 20% decrease in methane emissions, which they attributed to an increase in diet oil content from 20 g/kg DM to 51 g/kg DM due to the high oil content of mDDGS (127 g/kg DM). In the current study, however, methane output (g/day) was not affected by wDDGS inclusion level in either experiment and, although methane yield (g/kg DMI) differed among wDDGS inclusion levels in Experiment 1, variation was small and not systematic. It is likely that methane was not inhibited by highest inclusion levels of wDDGS because differences in dietary oil content were small, and any inhibition by wDDGS might have been offset by decreases in starch and increases in NDF content.

Indicators of cow health, such as plasma metabolites, rumination time, rumen pH and rumen VFA profile, were all within normal ranges and were not affected by wDDGS inclusion level, except for plasma urea-N and BHB in Experiment 1, and rumen isobutyrate in Experiment 2. Rumination time decreased when wDDGS replaced barley silage, but not when wDDGS replaced canola or barley grain [3], in agreement with the current study. Rumen pH was higher than anticipated for all diets, but rumen fluid samples collected by stomach tube are known to have a higher pH than samples collected through rumen cannulae. This is not due to saliva contamination during collection (the first litre of rumen fluid is discarded), but is due to the higher pH (+0.2–0.5) found in the cranial dorsal and cranial ventral regions compared to the central region of the rumen [27]. The lack of difference in rumen pH between diets is consistent with [28] who found no difference in rumen pH when wDDGS replaced up to 210 g/kg barley in beef finishing diets. Plasma urea-N was slightly above the normal range (3 to 5 mmol/L [29]), which is indicative of high ERDP relative to fermentable metabolisable energy. The lower ERDP content of wDDGS-1, compared to wDDGS-2, might explain why plasma urea-N decreased with increasing inclusion level in Experiment 1. Plasma BHB concentrations were below the threshold (1.0 mmol/L) for cows with subclinical ketosis [30]. In agreement with the current study, lower plasma BHB concentrations were observed in cows fed on diets with lower starch concentrations [31]. As in the current study, differences were not considered biologically important, and were attributed to lower propionate supply affecting hepatic capacity for fatty acid β-oxidation [31]. The decrease in rumen isobutyrate observed in Experiment 2 agrees with the findings of [7], who found that isobutyrate was the only VFA to change when canola meal was replaced by wDDGS at inclusion levels of up to 200 g/kg DM. Zhang [32] reported a decrease in isobutyrate when wDDGS replaced barley silage at 200 g/kg DM, and Mutsvangwa [33] reported a tendency for isobutyrate to decrease when wDDGS replaced canola meal at 120 and 157 g/kg DM. Chibisa [7] attributed the decrease in isobutyrate to a lower intake of branched-chain amino acids (BCAA) for cows fed on wDDGS, although [34] reported that the trend for decreased isobutyrate observed when a high-protein mDDGS replaced soya bean meal did not relate to amino acid composition of the protein supplements.

## 5. Conclusions

Results of Experiment 1 give confidence that wDDGS from bioethanol production can be included at approximately 200 g/kg DM in diets for high-yielding dairy cows without affecting feed intake, health, and milk yield. At an inclusion level of 240 g/kg DM, intake and milk yield were lower than control, but this might be attributed to the batch of wDDGS tested. This batch of wDDGS had a lower ME content than expected, probably due to a low proportion of solubles being added during manufacture. 

Results of Experiment 2 provide confidence that wDDGS can be included to at least 225 g/kg DM when a more typical batch of wDDGS is used. Results of Experiment 2 also suggest that balancing diets for minimum starch and saturated fatty acids, in addition to ME and MP supplies, might be beneficial for maintaining feed intake and milk yield of cows fed on wDDGS.

## Figures and Tables

**Table 1:** Ingredient composition ^1^ of partial-mixed rations containing four levels of wheat-based dried distillers’ grains with solubles (wDDGS).

Group	Experiment 1								Experiment 2			
	Early Lactation				Mid-Lactation							
wDDGS g/kg	0	80	160	240	0	80	160	240	0	75	150	225
Grass silage	171	172	173	175	186	187	189	190	185	186	188	188
Maize silage	262	264	266	268	286	288	289	291	208	209	211	212
Wheat silage	155	156	157	158	169	170	171	172	194	195	197	198
Wheat straw ^2^									21	21	22	22
wDDGS	0	97	195	295	0	94	188	285	0	68	220	271
SoyPass	0	7	14	21	0	6	11	17	0	9	29	36
Soya bean meal	105	71	36	0	104	70	35	0	87	66	16	0
Rapeseed meal	102	69	35	0	101	68	34	0	120	90	23	0
Wheat-rolled	142	109	76	42	89	59	30	0	109	93	57	45
Wheat-ground	22	22	22	22	23	24	24	24				
Molassed sugar beet pulp									44	33	8	0
Protected fat ^3^	12.3	8.3	4.2	0	12.2	8.2	4.1	0	11	11	11	11
Molasses	5.7	3.8	1.9	0	5.6	3.8	1.9	0				
Urea	2.3	1.5	0.8	0	2.3	1.5	0.8	0	3.5	2.6	0.7	0
Minerals and Vitamins ^4^	20	20	20	20	21	21	22	22	16	16	17	17

^1^ Values are g/kg on a dry matter basis; ^2^ for periods 3 and 4 in Experiment 1, chopped straw was added to each PMR at 0.48 kg DM/cow/day; ^3^ Golden Flake. ^4^ Composition (g DM/kg DM): limestone flour 301, sodium bicarbonate 241, Biotal Toxisorb 60, Biotal Binder 36, mineral and vitamin mix (containing: calcium, 17%; phosphorus, 6%; magnesium, 5%; salt, 17%; copper, 1250 mg/kg; manganese, 6250 mg/kg; cobalt, 100 mg/kg; zinc, 6000 mg/kg; iodine, 500 mg/kg; selenium, 25 mg/kg; vitamin A, 500,000 iu/kg; vitamin D3, 150,000 iu/kg; vitamin E, 3000 iu/kg and Biotin 133,000 mcg/kg) 361.

**Table 2:** Chemical composition of partial-mixed rations containing four levels of wheat-based dried distillers’ grains with solubles (wDDGS).

Group	Experiment 1 ^1^								Experiment 2			
	Early Lactation				Mid-Lactation							
wDDGS g/kg	0	80	160	240	0	80	160	240	0	75	150	225
DM, g/kg	453	452	451	450	434	433	433	432	442	441	440	440
	458	457	456	456	440	439	438	438				
Metabolisable energy, MJ/kg DM	12.2	12.2	12.2	12.2	12.1	12.1	12.0	12.0	11.7	11.7	11.8	11.8
	12.1	12.1	12.1	12.1	11.9	11.9	11.9	11.9				
Crude protein, g/kg DM	190	191	193	195	189	189	190	190	190	190	190	190
	186	188	190	191	185	186	186	187				
Starch, g/kg DM	218	202	186	169	201	187	172	158	296	272	218	200
	214	198	182	165	196	182	168	154				
Neutral-detergent fibre, g/kg DM	316	341	366	392	328	353	377	402	345	348	354	357
	327	351	376	402	340	363	387	412				
Oil-acid hydrolysis, g/kg DM	41.8	42.1	42.3	42.6	41.8	42.0	42.1	42.2	38.7	41.9	49.1	51.5
	41.1	41.3	41.6	41.9	41.0	41.2	41.3	41.4				

^1^ For each constituent in Experiment 1, the first row is for Periods 1 and 2 and the second row is for Periods 3 and 4 (with added straw). DM: Dry matter.

**Table 3:** (Experiment 1) Mean feed intake and performance of cows in early or mid-lactation when fed on diets containing 0 to 240 g wDDGS DM/kg diet DM.

Item	wDDGS Inclusion Level, g/kg						Linear Effect			Lactation Stage			
	0	80	160	240	SED	*p*	b	SE	*p*	Early	Mid	SED	*p*
Total DMI, kg/day	23.1 ^a^	23.1 ^a^	22.9 ^ab^	22.3 ^b^	0.31	0.029	−0.0026	0.0020	0.006	23.8	21.9	0.88	0.032
PMR DMI, kg/day	17.7 ^a^	17.8 ^a^	17.5 ^ab^	17.0 ^b^	0.27	0.025	−0.0032	0.0018	0.008	17.9	17.1	0.71	0.235
Conc DMI, kg/day	5.6	5.4	5.6	5.5	0.17	0.574	0.0006	0.0011	0.502	6.1	4.9	0.49	0.025
Milk yield, kg/day	43.5 ^a^	42.6 ^ab^	42.0 ^ab^	41.6 ^b^	0.69	0.009	−0.0107	0.0044	<0.001	47.2	37.8	2.32	0.003
ECM yield, kg/day	41.6 ^a^	40.0 ^ab^	39.5 ^ab^	38.7 ^b^	0.95	0.003	−0.0032	0.0018	0.008	43.1	36.9	2.11	0.017
Fat, kg/day	1.57 ^a^	1.49 ^ab^	1.44 ^ab^	1.41 ^b^	0.062	0.020	−0.0008	0.0004	0.002	1.52	1.43	0.103	0.442
Protein, kg/day	1.48 ^a^	1.45 ^ab^	1.43 ^ab^	1.42 ^b^	0.024	0.032	−0.0003	0.0001	0.004	1.61	1.29	0.077	0.003
Lactose, kg/day	1.98 ^a^	1.92 ^ab^	1.91 ^ab^	1.89 ^b^	0.032	0.040	−0.0005	0.0002	0.004	2.15	1.70	0.104	<0.001
Fat, g/kg	37.4	35.7	34.9	34.9	1.37	0.167	−0.0076	0.0086	0.034	33.1	38.3	2.07	0.007
Protein, g/kg	34.0	34.1	34.1	34.2	0.14	0.314	0.0003	0.0009	0.062	34.2	34.0	0.22	0.444
Urea, mg/dL	38.4 ^a^	37.3 ^a^	34.3 ^b^	33.5 ^b^	1.05	<0.001	−0.0192	0.0067	<0.001	37.6	34.2	1.10	0.015
Lactose, g/kg	45.4	45.5	45.6	45.6	0.22	0.664	0.0004	0.0010	0.213	45.7	45.4	0.29	0.349
Live weight, kg	672	670	670	671	2.2	0.352	−0.0063	0.0145	0.435	670	671	22.9	0.963
BCS	2.60	2.51	2.56	2.56	0.054	0.235	−0.0003	0.0003	0.842	2.56	2.56	0.240	0.986
Methane, g/day	360	359	363	359	2.7	0.261	0.0038	0.0174	0.787	361	360	5.5	0.835
Methane, g/kg DMI	15.9 ^ab^	15.9 ^a^	16.2 ^ab^	16.6 ^b^	0.31	0.022	0.0018	0.0020	0.005	15.4	16.9	0.93	0.156
Rumination, min/day	496	496	488	485	8.1	0.322	−0.0398	0.0521	0.164	520	462	27.5	0.063
DM digestibility	0.713	0.714	0.703	0.705	0.011	0.693	−0.00004	−0.00006	0.396	0.712	0.706	0.009	0.534
N digestibility	0.708 ^a^	0.707 ^a^	0.681 ^ab^	0.674 ^b^	0.013	0.017	−0.00016	0.00007	0.002	0.697	0.688	0.010	0.453

wDDGS, wheat-based dried distillers’ grains with solubles; SED, standard error of difference for comparing group means; *p*, F-ratio or linear effect probability; b, linear effect of wDDGS inclusion level; SE, standard error of linear effect; DMI, dry matter intake; PMR, partial-mixed ration; Conc, concentrate fed during milking; ECM, energy-corrected milk; BCS, body condition score; DM, dry matter; N, nitrogen; a,b means in same row with different superscripts differ (*p* < 0.05).

**Table 4:** (Experiment 1) Mean plasma metabolites of cows in early or mid-lactation when fed on diets containing 0 to 240 g wDDGS DM/kg diet DM.

Item	wDDGS Inclusion Level, g/kg						Linear Effect			Lactation Stage			
	0	80	160	240	SED	*p*	b	SE	*p*	Early	Mid	SED	*p*
Albumin, g/L	26.5	24.7	26.1	26.3	0.97	0.394	0.0002	0.00630	0.612	26.2	25.6	0.74	0.418
Globulin, g/L	27.5	26.4	26.6	26.8	1.04	0.496	−0.0064	0.00678	0.781	26.7	27.0	1.67	0.830
Total Protein, g/L	54.0	51.0	52.7	53.1	1.56	0.236	−0.0075	0.01012	0.927	52.8	52.5	1.84	0.898
Urea-N, mmol/L	5.4 ^a^	5.2 ^ab^	5.0 ^bc^	4.8 ^c^	0.13	<0.001	−0.0029	0.00084	<0.001	5.3	4.9	0.17	0.009
BHB, mmol/L	0.53 ^a^	0.52 ^a^	0.65 ^b^	0.60 ^b^	0.034	<0.001	0.0006	0.00023	0.003	0.56	0.60	0.040	0.363
NEFA, mmol/L	0.45	0.36	0.39	0.38	0.047	0.409	−0.0004	0.00030	0.360	0.44	0.35	0.041	0.034
Glucose, mmol/L	2.56	2.69	2.58	2.67	0.121	0.539	0.0003	0.00078	0.632	2.62	2.64	0.083	0.778

wDDGS, wheat-based dried distillers’ grains with solubles; SED, standard error of difference for comparing group means; *p*, F-ratio or linear effect probability; b, linear effect of wDDGS inclusion level; SE, standard error of linear effect; BHB, β-hydroxybutyrate; NEFA, non-esterified fatty acids; a,b,c means in same row with different superscripts differ (*p* < 0.05).

**Table 5:** (Experiment 2) Mean feed intake and performance of cows when fed on diets containing 0 to 225 g wDDGS DM/kg diet DM.

Item	wDDGS Inclusion Level, g/kg						Linear Effect		
	0	75	150	225	SED	*p*	b	SE	*p*
DMI, kg/day	22.9	22.5	22.2	22.1	0.36	0.188	−0.0033	0.00156	0.034
PMR DMI, kg/day	18.0	17.7	17.4	17.7	0.35	0.376	−0.0018	0.00149	0.233
Conc DMI, kg/day	4.8	4.8	4.8	4.4	0.21	0.223	−0.0016	0.00092	0.081
Milk yield, kg/day	32.6	32.8	32.2	32.1	0.64	0.3332	−0.0049	0.00271	0.070
ECM yield, kg/day	32.7	33.2	32.4	32.1	0.69	0.408	−0.0035	0.00295	0.233
Fat, kg/day	1.25	1.30	1.26	1.25	0.037	0.500	−0.00006	0.00016	0.704
Protein, kg/day	1.14	1.14	1.11	1.11	0.022	0.182	−0.0002	0.00009	0.058
Lactose, kg/day	1.47	1.47	1.47	1.45	0.038	0.764	−0.0001	0.00016	0.542
Fat, g/kg	39.1	40.7	40.7	40.1	0.89	0.195	0.0038	0.00383	0.323
Protein, g/kg	35.1	35.4	35.0	35.0	0.35	0.598	−0.0009	0.00149	0.538
Urea, mg/dL	37.8 ^a^	35.9 ^b^	35.6 ^bc^	33.9 ^c^	0.87	<0.001	−0.0153	0.00370	<0.001
Lactose, g/kg	45.2	45.2	45.4	45.4	0.16	0.111	0.0016	0.00069	0.053
Live weight, kg	712	710	705	707	3.0	0.102	−0.0268	0.01295	0.061
BCS	3.22	3.19	3.22	3.18	0.051	0.685	−0.0002	0.00022	0.406
Methane, g/day	432	436	423	434	7.1	0.292	−0.0107	0.03077	0.728
Methane, g/kg DMI	19.4	19.9	19.7	19.8	0.60	0.784	0.0017	0.0025	0.509
Rumination, min/day	441	430	427	426	8.0	0.231	−0.0652	0.03428	0.060
DM digestibility	0.769	0.772	0.765	0.770	0.0078	0.322	−0.00005	0.00003	0.100
N digestibility	0.725	0.734	0.721	0.718	0.0107	0.458	−0.00005	0.00004	0.274

wDDGS, wheat-based dried distillers’ grains with solubles; SED, standard error of difference for comparing group means; *p*, F-ratio or linear effect probability; b, linear effect of wDDGS inclusion level; SE, standard error of linear effect; DMI, dry matter intake; PMR, partial-mixed ration; Conc, concentrate fed during milking; ECM, energy-corrected milk; BCS, body condition score; DM, dry matter; N, nitrogen; a,b,c means in same row with different superscripts differ (*p* < 0.05).

**Table 6:** (Experiment 2) Mean plasma metabolites of cows when fed on diets containing 0 to 225 g wDDGS DM/kg diet DM.

Item	wDDGS Inclusion Level, g/kg						Linear Effect		
	0	75	150	225	SED	*p*	b	SE	*p*
Albumin, g/L	34.3	33.8	33.7	34.6	0.77	0.608	0.0010	0.00328	0.760
Globulin, g/L	39.9	40.4	41.2	41.0	1.34	0.748	0.0057	0.00567	0.314
Total Protein, g/L	74.2	74.2	74.8	75.6	0.99	0.435	0.0065	0.00420	0.123
Urea-N, mmol/L	5.53	5.35	5.35	5.20	0.15	0.176	−0.0013	0.00062	0.036
BHB, mmol/L	0.55	0.55	0.52	0.52	0.031	0.739	−0.0001	0.00013	0.292
NEFA, mmol/L	0.08	0.10	0.09	0.10	0.011	0.236	0.00005	0.00005	0.276
Glucose, mmol/L	3.67	3.61	3.66	3.70	0.045	0.299	0.0002	0.00019	0.382

wDDGS, wheat-based dried distillers’ grains with solubles; SED, standard error of difference for comparing group means; *p*, F-ratio or linear effect probability; b, linear effect of wDDGS inclusion level; SE, standard error of linear effect; BHB, β-hydroxybutyrate; NEFA, non-esterified fatty acids.

**Table 7:** (Experiment 2) Mean rumen pH and volatile fatty acid concentrations of cows when fed on diets containing 0 to 225 g wDDGS DM/kg diet DM.

Item	wDDGS Inclusion Level, g/kg						Linear Effect		
	0	75	150	225	SED	*p*	b	SE	*p*
Rumen pH	6.72	6.67	6.71	6.73	0.053	0.621	0.0001	0.00023	0.679
Acetate, mmol/L	72.9	71.9	70.9	66.7	3.21	0.241	−0.0263	0.01359	0.055
Propionate, mmol/L	29.5	29.7	29.5	27.9	1.75	0.693	−0.0070	0.00744	0.347
Butyrate, mmol/L	17.6	17.8	17.9	17.2	1.06	0.904	−0.0017	0.00447	0.704
Isobutyrate, mmol/L	1.42 ^a^	1.41 ^a^	1.31 ^ab^	1.23 ^b^	0.06	0.007	−0.0009	0.00027	<0.001
Isovalerate, mmol/L	1.83	1.81	1.72	1.59	0.10	0.096	−0.0011	0.00044	0.015
Valerate, mmol/L	1.90	1.96	2.05	1.98	0.14	0.768	0.0004	0.00059	0.463
Total VFA, mmol/L	125	125	123	117	6.0	0.438	−0.0370	0.02538	0.148

wDDGS, wheat-based dried distillers’ grains with solubles; SED, standard error of difference for comparing group means; *p*, F-ratio or linear effect probability; b, linear effect of wDDGS inclusion level; SE, standard error of linear effect; VFA, volatile fatty acids; a,b means in same row with different superscripts differ (*p* < 0.05).

## Data Availability

The data presented in this study are available on request from the corresponding author. The data are not publicly available due to restrictions of the ENBBIO collaboration agreement.

## Appendix A

### Appendix A.1. Metabolisable Energy Content of wDDGS

#### Appendix A.1.1. Materials and Methods

Metabolisable energy (ME) contents of the two wDDGS samples used in Experiments 1 and 2 were determined in sheep fed at the maintenance level of feeding. Grass hay (chopped) was used as the basal forage. Composition of hay and wDDGS samples is shown in Table A1. The same eight wether sheep (live weight 50–60 kg) were used throughout the metabolism study. Sheep were fed twice daily at approximately 08:30 and 16:00. Water was available ad libitum.

Each ME determination comprised three periods lasting 26 days each. In the first period, sheep were fed on hay (1 kg/day in two equal meals). In the second period, sheep were fed on hay (700 g/day) and wDDGS (700 g/day) in two equal meals. In the third period, sheep were fed hay (1 kg/day in two equal meals). During the first 16 days of each period, sheep were housed in individual pens, bedded on hemp; for the remaining 10 days, sheep were housed in individual metabolism crates.

Whilst in metabolism crates, any feed refusals were weighed and recorded. Faecal output was weighed twice daily and a subsample of approximately 200 g taken for analysis. Urine was collected into a plastic tub and volumes measured twice daily using a measuring cylinder. Evaporation of volatiles such as ammonia was prevented by prior acidification of urine tubs with 20 mL of 50% sulphuric acid. A urine subsample of approximately 150 mL was taken twice daily for analysis. Faecal and urine samples were stored at −20 °C until analysed.

Samples of feed, faeces and urine were analysed for DM, nitrogen and gross energy (GE) contents. Dry matter was determined by oven drying at 80 °C until stable weight (usually around five days). Nitrogen was determined by elemental Dumas analyser (Leco Truspec Analyser; Leco Instruments, Stockport, UK). Gross energy was determined by bomb calorimeter.

Methane output was measured using respiration chambers, as described by Garnsworthy et al. [15]. Due to the volume and airflow of the chambers, four sheep had to be in a chamber at the same time, to generate sufficient methane for accurate measurement. The eight sheep were therefore divided into two batches of four sheep. For each wDDGS sample studied, methane measurements were made in three periods, each lasting 14 days. During the first seven days of each period, sheep were housed in individual pens in a barn; for the remaining seven days, sheep were housed in individual pens within a respiration chamber. In Periods 1 and 3, each sheep was fed on hay (1 kg/day in two equal meals); in Period 2, each sheep was fed on hay (700 g/day) and wDDGS (700 g/day) in two equal meals. Methane concentration was measured at the inlet and outlet vents of the respiration chamber. Air was sampled alternately from inlet and outlet for periods of two minutes, with methane concentration recorded for the second minute of each period. Methane production in each four-minute sampling period was calculated as the difference between methane concentration at inlet and outlet multiplied by airflow in the outlet duct. Data for 32 min after the door was opened at each feeding time were discarded. Methane production was calculated as average daily emission rate, rate per kg DMI, and as a percentage of daily GE intake.

Digestibility coefficients for DM, nitrogen and GE were calculated for each day of the collection periods in metabolism crates as:

Digestibility = (Intake − Faecal Output)/Intake

where Intake is the sum of feed components offered at am and pm meals minus any refusals, and Faecal Output is the sum of faecal components recorded at am and pm weighings.

In Period 2, faecal output attributed to wDDGS was calculated as:

Faecal Output from wDDGS = Total Faecal Output − [Hay Intake × (1 − Hay Digestibility)]

where Total Faecal Output is the sum of faecal components recorded at am and pm weighings, Hay Intake is the sum of hay components offered at am and pm meals minus any refusals, and Hay Digestibility is the average digestibility of hay components in Periods 1 and 3 for each individual sheep.

In Period 2, urine output attributed to wDDGS was calculated as:

Urine GE Output from DDGS = Total Urine GE Output − (Hay GE Intake × Hay GE Urine)

where Hay GE Urine is the average proportion of Hay GE Intake lost in urine in Periods 1 and 3 for each individual sheep.

Metabolisable energy content of feeds was calculated as:

ME (g/kg DM) = (GE Intake − Faecal GE Output − Urine GE Output − CH_4_ GE Output)/DMI

where CH_4_ GE Output is methane energy output calculated as GE Intake × Methane Factor. Methane Factor is the percentage of GE intake lost as CH_4_ for each diet, measured in a respiration chamber.

Data were analysed using the residual maximum likelihood (REML) procedure of Genstat (19th Edition; VSN International, Hemel Hempstead, UK). For digestibility and ME data, the fixed effect in the model was wDDGS, and random effects were individual sheep and day of sampling. For methane data, the fixed effect in the model was wDDGS, and random effects were batch of sheep and day of sampling.

#### Appendix A.1.2. Results

**Table A1:** Composition of grass hay, wDDGS-1 and wDDGS-2.

	Grass Hay	wDDGS-1	wDDGS-2
Dry matter (DM), g/kg	866	884	895
Gross energy, MJ/kg DM	17.3	21.4	20.9
Crude protein, g/kg DM	80	312	361
Neutral detergent fibre, g/kg DM	602	352	394
Ether extract, g/kg DM	17	76	46
Ash, g/kg DM	69	44	59

**Table A2:** Digestibility coefficients and metabolisable energy (ME) content of wDDGS-1 and wDDGS-2.

	wDDGS-1	wDDGS-2	SED	*p*
Digestibility				
Dry matter (DM)	0.649	0.703	0.0208	0.012
Nitrogen	0.715	0.733	0.0121	0.150
Energy	0.676	0.715	0.0190	0.004
ME (MJ/kg DM)	12.1	13.4	0.42	0.002

SED, standard error of difference for comparing means; *p*, F-ratio probability.

**Table A3:** Methane output by sheep fed on diets consisting of hay plus wDDGS-1 or wDDGS-2.

	wDDGS-1	wDDGS-2	SED	*p*
Methane, g/day	14.9	10.1	0.46	<0.001
Methane, g/kg DMI	10.7	7.22	0.33	<0.001
Methane, % GEI	3.01	2.17	0.094	<0.001

SED, standard error of difference for comparing means; *p*, F-ratio probability; GEI, Gross energy intake (MJ/day).

### Appendix A.2. Rumen Degradability of wDDGS

#### Appendix A.2.1. Materials and Methods

Rumen degradability of protein and dry matter in wDDGS samples were determined by a synthetic fibre bag technique based upon the method of Ørskov and McDonald [35]. Bags were incubated in the rumen of two non-lactating Holstein–Friesian dairy cows fed on grass hay (5 kg/day) and concentrates (2 kg/day).

For each sample, approximately 20 g of DDGS was weighed accurately into each of 28 synthetic fibre bags. Fourteen bags were inserted into the rumen of each cow and removed after 0, 4, 8, 12, 24, 48 or 72 h (two bags per cow per incubation time). After removal, synthetic fibre bags were rinsed immediately under running water and then washed in a domestic washing machine for 20 min at 30 °C. Bags were then placed in an oven at 80 °C for a minimum of 48 h or until a constant weight was achieved on two consecutive days. Residues remaining in the Dacron bags were analysed for nitrogen content using an elemental analyser (Dumas method). Aqueous solubility of dry matter (DM) and nitrogen in the original samples were determined by repeated washing and filtering through a Whatman 541 filter paper.

Loss of DM and nitrogen from bags for each sample was fitted by non-linear regression to the model:

D = *a* + *b* (1 − e^−*ct*^)

where D = disappearance of DM or N at time *t*, *a* is the intercept which represents the rapidly soluble fraction, *b* is the asymptote which represents the potentially degradable fraction, and *c* is the exponential rate of degradation.

Effective degradability (ED) was calculated using the equation

$$\mathrm{Effective}\;\mathrm{degradability}\;=a+\frac{bc}{\left(c+k\right)}$$

where *k* is the fractional outflow from the rumen, assumed for dairy cows to be 0.08 (8% per hour).

Effective rumen degradable protein (ERDP) was calculated using the equation

ERDP = CP × ED

where CP is crude protein content of wDDGS.

Digestible undegradable protein (DUP) was calculated using the equation

DUP = 0.9(CP − ERDP) − (6.25 × ADIN)

where ADIN is acid detergent insoluble nitrogen, which was assumed to be 7.0 g/kg DM, as shown in the Feed into Milk database [9].

#### Appendix A.2.2. Results

**Table A4:** Rumen dry matter (DM) and nitrogen (N) degradability parameters of wDDGS-1 and wDDGS-2.

	WDDGS-1		wDDGS-2	
	DM	N	DM	N
Solubility	0.352	0.185	0.335	0.255
a	0.510	0.236	0.334	0.311
b	0.361	0.670	0.402	0.464
c	0.044	0.039	0.100	0.099
Degradability	0.638	0.455	0.557	0.568
ERDP, g/kg DM		142		205
DUP, g/kg DM		109		96

a, b, c are parameters of the degradability model. ERDP = effective rumen degradable protein at rumen outflow rate of 0.08. DUP = digestible undegradable protein.
